# Health effects of routine measles vaccination and supplementary immunisation activities in 14 high-burden countries: a Dynamic Measles Immunization Calculation Engine (DynaMICE) modelling study

**DOI:** 10.1016/S2214-109X(23)00220-6

**Published:** 2023-07-18

**Authors:** Megan Auzenbergs, Han Fu, Kaja Abbas, Simon R Procter, Felicity T Cutts, Mark Jit

**Affiliations:** a Department of Infectious Disease Epidemiology, London School of Hygiene & Tropical Medicine, London, UK; b Public Health Foundation of India, New Delhi, India; c School of Public Health, University of Hong Kong, Hong Kong Special Administrative Region, China

## Abstract

**Background:**

WHO recommends at least 95% population coverage with two doses of measles-containing vaccine (MCV). Most countries worldwide use routine services to offer a first dose of measles-containing vaccine (MCV1) and later, a second dose of measles-containing vaccine (MCV2). Many countries worldwide conduct supplementary immunisation activities (SIAs), offering vaccination to all people in a specific age range irrespective of previous vaccination history. We aimed to estimate the relative effects of each dose and delivery route in 14 countries with high measles burden.

**Methods:**

We used an age-structured compartmental dynamic model, the Dynamic Measles Immunization Calculation Engine (DynaMICE), to assess the effects of different vaccination strategies on measles susceptibility and burden during 2000–20 in 14 countries with high measles incidence (containing 53% of the global birth cohort and 78% of the global measles burden). Country-specific routine MCV1 and MCV2 coverage data during 1980–2020 were obtained from the WHO and UNICEF Estimates of National Immunization Coverage database for all modelled countries and SIA data were obtained from the WHO summary of measles and rubella SIAs. We estimated the incremental health effects of different vaccination strategies using prevented cases of measles and deaths from measles and their efficiency using the incremental number needed to vaccinate (NNV) to prevent an additional measles case.

**Findings:**

Compared with no vaccination, MCV1 implementation was estimated to have prevented 824 million cases of measles and 9·6 million deaths from measles, with a median NNV of 1·41 (IQR 1·35–1·44). Adding routine MCV2 to MCV1 was estimated to have prevented 108 million cases and 404 270 deaths, whereas adding SIAs to MCV1 was estimated to have prevented 256 million cases and 4·4 million deaths. Despite larger incremental effects, adding SIAs to MCV1 (median incremental NNV 6·02, 5·30–7·68) showed reduced efficiency compared with adding routine MCV2 (5·41, 4·76–6·11).

**Interpretation:**

Vaccination strategies, including non-selective SIAs, reach a greater proportion of children who are unvaccinated and reduce measles burden more than MCV2 alone, but efficiency is lower because of the wide age range targeted by SIAs. This analysis provides information to help improve the health effects and efficiency of measles vaccination strategies. The interplay between MCV1, MCV2, and SIAs should be considered when planning future measles vaccination strategies.

**Funding:**

Gavi, the Vaccine Alliance and the Bill & Melinda Gates Foundation.

## Introduction

Between 2000 and 2020, deaths from measles were estimated to have decreased by 94% globally,^1^ which was mostly achieved through routine immunisation and supplementary immunisation activities (SIAs) with measles-containing vaccines (MCVs).^2^, ^3^, ^4^, ^5^ According to WHO, the first routine dose of measles-containing vaccine (MCV1) should be given during the first year of life, ideally at age 9 months or age 12 months. The second routine dose of measles-containing vaccine (MCV2) is recommended to be given between age 15 months and 18 months. SIAs are vaccination campaigns that deliver vaccine doses using strategies other than via routine services and are usually non-selective (ie, vaccination is offered irrespective of vaccination history). Throughout this Article, the term SIA indicates non-selective SIAs.

Since the introduction of measles vaccination in high-income countries in the 1960s and in low-income and middle-income countries in the 1970s and 1980s, recommendations around measles vaccination strategies have been revised. Historically, low-income and middle-income countries relied on MCV1 with SIAs to interrupt transmission and reach children who were unvaccinated. In 2009, WHO recommended introducing MCV2 once a country reached 80% MCV1 coverage, retaining an emphasis on aiming for high coverage with MCV1 as soon as possible after a child loses antibodies from the birthing parent. In 2017, this recommendation was revised to state that countries should include MCV2 in routine immunisation schedules regardless of MCV1 coverage. Furthermore, operational support to strengthen routine immunisation infrastructure when incorporating MCV2 should be provided. Partly due to concerns about the sustainability of funding for nationwide, non-selective SIAs and their potential to disrupt routine services,^6^, ^7^ WHO has proposed that such SIAs can be phased out once countries have more than 95% coverage of both routine doses.^8^

**Research in context**

**Evidence before this study**

We searched PubMed for manuscripts published in English between Jan 1, 2000, and March 10, 2022, that contained the following search terms: (“measles” or “MCV” or “MCV1” or “MCV2”) and (“vaccin*” or “immun*”) and (“supplementary immun* activit*” or “campaign” or “catch-up”) and “model”. 13 modelling studies investigating vaccination strategies to effectively control measles were identified and were included in a risk-of-bias assessment. Although several articles recommended that sustaining a high coverage of routine immunisation and campaigns was optimal for measles control, we found only three studies that explicitly addressed the interactions between different delivery strategies of measles-containing vaccine (MCV) doses. One modelling study concluded that, in Zambia, a second routine dose of measles-containing vaccine (MCV2) as a vaccination strategy can sustain high levels of population immunity and that frequent, low-coverage supplementary immunisation activities (SIAs) might sustain higher levels of immunity than less frequent, high-coverage SIAs. However, direct comparisons of the incremental differences between strategies were not conducted. The second modelling study assessed the vaccination effects of incrementally introducing the first routine dose of measles-containing vaccine (MCV1), MCV2, and SIAs compared with a counterfactual scenario without measles vaccination, but also did not directly compare MCV2 with SIAs. The third modelling study showed that in addition to MCV1, delivering MCV2 was more cost-effective and prevented more cases of measles than SIAs in a hypothetical cohort in DR Congo.

**Added value of this study**

We analysed the relative roles of MCV1, MCV2, and SIAs in preventing measles transmission during 2000–20 and assessed the incremental health effects of historical vaccination policies implemented in high-burden countries. We also assessed the efficiency of SIAs compared with routine immunisation in terms of the number needed to vaccinate between comparative scenarios. As many countries rebuild their health systems after the COVID-19 pandemic, quantifying the incremental effects of different vaccination strategies that have historically been implemented is useful, as is understanding the incremental effects and efficiency of each strategy to minimise measles burden and maximise the reach of vaccines to children who are unvaccinated and under-vaccinated. Furthermore, as countries continue to introduce MCV2, in principle, reliance on non-selective SIAs should decrease and eventually stop once high population immunity (ie, >93%) can be maintained with a routine two-dose schedule alone. The novelty of our study is the direct comparison of historical vaccination strategies implemented at different times across high-burden countries, rather than comparing only hypothetical scenarios about coverage.

**Implications of all the available evidence**

Our results show that in many high-burden countries, SIAs are better at reaching children who have not received any doses of MCV, prevent more cases of measles and deaths from measles than MCV2, and effectively reduce measles-outbreak potential by keeping the size of the susceptible population smaller than the size of a birth cohort. To achieve high levels of vaccination coverage and meet targets for measles elimination in high-burden areas, SIAs should be strengthened; they could be made more efficient and designed to fit local demand until countries can achieve very high two-dose routine coverage.

The implementation of SIAs over time has been motivated by different goals and needs. SIAs were a major component of the measles elimination strategy implemented widely in the Americas in the 1990s, with high routine MCV1 coverage and occasional follow-up SIAs sustaining elimination since July, 2015.^9^, ^10^ In other regions, such as Africa and southeast Asia, SIAs have increased population immunity in countries with low MCV1 or MCV2 coverage. In these countries, SIAs have been a highly effective and equitable strategy for protecting hard-to-reach children who would otherwise be missed by routine immunisation,^11^, ^12^ although the relative reach of SIAs versus routine immunisation varies between and within countries.^13^ To prevent measles transmission and subsequent outbreaks, a commonly used criterion is that a follow-up SIA should be conducted before the cumulative number of susceptible children younger than 5 years approaches the size of a birth cohort (including the newborn population of a year).^14^, ^15^ Historically, this criterion has been influential in informing the timing of SIAs so the number of susceptible children remains less than the size of one birth cohort and measles transmission can be interrupted and elimination can be achieved.^8^ In practice, even if countries recognise that a follow-up SIA is due and correctly identify the age groups with the highest prevalence of susceptibility, delays in obtaining funding or competing priorities, such as other pathogens, might lead to delayed implementation of an SIA or a narrower than ideal age range targeted, which reduces the effects of the SIA.^15^ Outbreak-response SIAs might then be needed, the effects of which depend on the speed of response, geographical extent, and coverage attained.^16^ Many countries, therefore, have implemented a mixture of so-called preventive campaigns targeting various age groups at national or subnational levels and reactive campaigns that aim to shorten outbreaks.

In 2012, the World Health Assembly endorsed the Global Vaccine Action Plan, which included a commitment to achieving measles elimination in five of the six WHO regions by 2020. During 2000–10, estimated global MCV1 coverage increased from 72% to 84%, but has since stagnated. However, estimated routine MCV2 coverage has increased from 18% in 2000 to 70% in 2020.^17^ In this retrospective analysis of measles vaccination policies during 2000–20, we aimed to use the Dynamic Measles Immunization Calculation Engine (DynaMICE), a population-based dynamic model of measles transmission, to better understand the effects of different vaccination strategies that have been used in 14 high-burden countries.

## Methods

### Data sources

Reported measles cases, collected through the WHO and UNICEF Joint Reporting Form on Immunization, and estimated measles incidence data from the Institute for Health Metrics and Evaluation (IHME), were used to obtain separate rankings of countries by measles incidence from 2010 to 2019.^18^, ^19^ We included the ten countries with the highest incidence from each data source (appendix p 1), which resulted in 14 countries being included in the analysis (ie, India, Nigeria, Indonesia, Ethiopia, China, Philippines, Uganda, DR Congo, Pakistan, Angola, Madagascar, Ukraine, Malawi, and Somalia). These countries contained 53% of the global birth cohort and 78% of the global measles burden.

Country-specific routine MCV1 and MCV2 coverage data during 1980–2020 were obtained from the WHO and UNICEF Estimates of National Immunization Coverage (WUENIC) database for all modelled countries^20^ and SIA data were obtained from the WHO summary of measles and rubella SIAs (appendix pp 2–6).^21^ Year of MCV2 introduction varied between countries (appendix p 1). We extracted the start and end dates of SIA implementation, targeted age group, and number of doses given during each SIA. Knowing whether the entire country was covered after phased or subnational SIAs was not always possible, so we calculated country-level coverage for each SIA by comparing reported SIA doses with the national population in the target age range from World Population Prospects 2019.^22^

### DynaMICE model

DynaMICE is an age-structured compartmental model of measles transmission that considers time-varying states of disease (ie, maternally immune or immune from birthing parent, susceptible, infectious, or recovered) and vaccination (ie, no doses, one dose, two doses, or three or more doses). The model has been used previously for estimating the effects of measles vaccination^2^, ^7^ and a description of the model structure, parameters, and equations has been published.^23^ Using the DynaMICE model, we modelled country-level routine immunisation programmes on the basis of historical WUENIC coverage estimates for MCV1 and MCV2^19^ following nationally recommended schedules (appendix p 1). We modelled each SIA according to the target age group and median date of implementation in each country in the WHO record (appendix pp 2–6). We assumed that SIA doses are more likely to reach children who had been previously vaccinated by distributing doses randomly among the target population, except for a proportion who are less likely to be reached by current childhood vaccination programmes. This population who are less likely to be reached could only be covered by a campaign when all the other target populations have received an SIA dose. This proportion of children who are less likely to be reached was assumed to be 7·7% of the total country-level population in each country on the basis of the population-weighted mean estimate for children aged 1 year who were missing all diphtheria–pertussis–tetanus, BCG, measles-containing, and polio vaccines in 92 low-income and middle-income countries during 2010–19 (appendix p 10).^24^ In the DynaMICE model, MCV1 efficacy increases linearly by 1·49% per increased month of age, resulting in 78% efficacy for children aged 9 months and 82% efficacy for children aged 12 months.^20^, ^25^ MCV2 efficacy depends on the level of MCV1 protection (ie, the proportion of vaccinated people who are effectively protected) received previously, and two-dose vaccine efficacy is capped at 98%.^20^, ^26^ The basic reproduction number (R_0_) of measles was 15·9 based on a summary estimate taken from endemic settings.^27^ Country-dependent and age-dependent social-contact matrices^28^ were used to inform the country-specific patterns of measles transmission. To include epidemic patterns since the global implementation of MCV, the model simulation began in 1980.

### Measles vaccination strategies and effect estimates

Using the DynaMICE model, we assessed cases of measles and deaths from measles during 2000–20 across the following vaccination strategies: no vaccination; MCV1 alone; MCV1 and MCV2; MCV1 and SIAs; and MCV1, MCV2, and SIAs. We estimated deaths by multiplying the model estimates of cases of measles with age-specific, year-specific, and country-specific case-fatality ratios;^29^ the model did not account for non-measles-specific vaccine effects on preventing deaths. We calculated the annual incidence of measles per 1 million population and compared the susceptible population of children younger than 5 years with the birth cohort, defined as the mid-year population aged 0–1 year, to understand the potential of different delivery strategies to reduce transmission and outbreaks. To estimate the incremental effects of historical measles vaccine strategies, each strategy was compared with a counterfactual strategy that was representative of a historical policy decision for measles vaccination. MCV1 was compared with the alternative of no vaccination, whereas the MCV1 and MCV2 strategy and the MCV1 and SIAs strategy were compared with MCV1 alone. The MCV1, MCV2 and SIAs strategy was compared with the counterfactual strategy of MCV1 and SIAs, as well as separately compared with the MCV1 and MCV2 strategy. Although the same comparator strategies were evaluated across countries, countries adopted varying policies, such as year of MCV2 introduction or frequency of SIAs. For each strategy, historical coverage data were used, and for each pair of comparisons we estimated the health effects of an additional delivery strategy by calculating the cumulative vaccine-prevented cases and deaths, and the efficiency of adding a delivery strategy by calculating the incremental number of doses needed to vaccinate (NNV) to prevent an additional measles case during 2000–20.

### Sensitivity analysis

We modelled vaccine effects if MCV2 had been introduced in 2000 under fast or gradual roll-out (appendix p 9). For each year during 2000–20, we assumed that the alternative MCV2 coverage was either 10% lower than the MCV1 coverage of each country or equal to the MCV2 coverage of each country in that year, whichever was larger. Moreover, we modelled two alternative assumptions about the likelihood of receipt of an SIA dose according to past vaccination history (appendix p 10). In this sensitivity analysis, we defined the so-called zero-dose population as children receiving no MCV doses. One assumption is that SIA doses preferentially reach children who are already vaccinated and that any remaining doses after all children who are already vaccinated are reached are then given to children in the zero-dose population. However, the other assumption is that a strategy reaches children in the zero-dose population first, and the remaining doses are then given to children who are already vaccinated.

### Role of the funding source

The funders had no role in study design, data collection, data analysis, data interpretation, or writing of the report.

## Results

**Figure 1:**
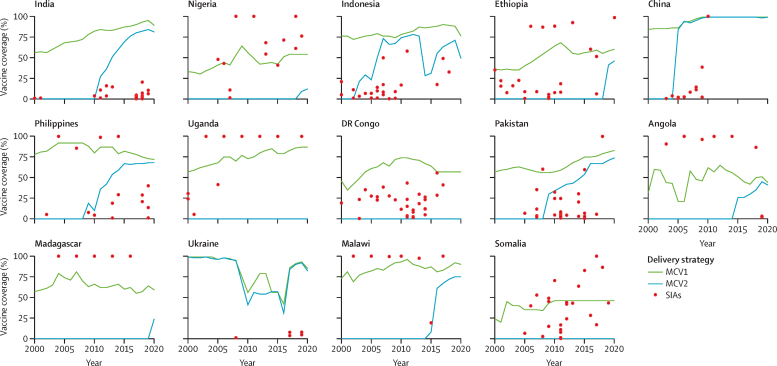
Immunisation coverage for MCV1, MCV2, and SIAs (2000–20) SIA coverage was calculated from reported numbers of doses administered and national populations in the SIA target age group. As of 2020, Uganda, DR Congo, and Somalia had not implemented MCV2. Additional years of MCV2 introduction for other countries are available (appendix p 1). MCV1=the first routine dose of measles-containing vaccine. MCV2=the second routine dose of measles-containing vaccine. SIA=supplementary immunisation activity.

Between the 14 analysed countries, there were notable differences in MCV1 and MCV2 coverage during 2000–20 (figure 1)—some countries had not introduced MCV2 as of 2020 (ie, Uganda, Somalia, and DR Congo) or introduced MCV2 much later in time (ie, Madagascar, Ethiopia, Nigeria, and Angola). Comparatively, some countries introduced MCV2 early and sustained high coverage (ie, India, Pakistan, and China), whereas in others, MCV2 coverage fluctuated over time (ie, Indonesia and Ukraine).

**Figure 2:**
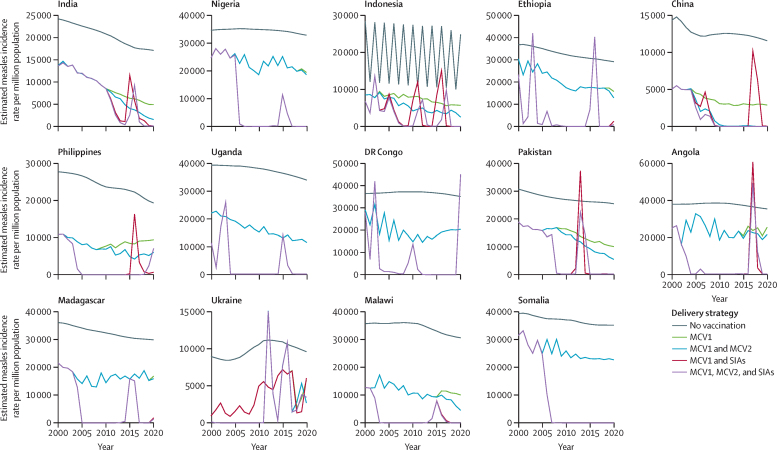
Estimated annual measles incidence rate per million population across different vaccination delivery strategies (2000–20) Temporal trends in measles incidence rates vary by different vaccination delivery strategies; the measles burden decreases with additional vaccination delivery strategies. For countries that have not yet introduced MCV2 (ie, Uganda, DR Congo, and Somalia), there are overlapping trends for incidence rates for the delivery strategies of MCV1 and MCV2 (blue lines) and MCV1 only (green lines) and the delivery strategies of MCV1, MCV2, and SIAs (purple lines) and MCV1 and SIAs (red lines). Overlapping trends are also seen in most analysed years in countries that introduced MCV2 after 2017 (ie, Nigeria, Ethiopia, and Madagascar). In Indonesia, the fluctuations seen in the no vaccination strategy are the result of dynamic sizes of the susceptible population affected by natural seasonality of measles transmission. MCV1=the first routine dose of measles-containing vaccine. MCV2=the second routine dose of measles-containing vaccine. SIA=supplementary immunisation activity.

Compared with the no vaccination strategy, measles incidence rates declined substantially in all 14 high-burden countries in strategies with MCV1 only (figure 2). In the strategy in which MCV1 and MCV2 were used without SIAs, the annual burden of measles declined slowly over time and endemic transmission continued. With MCV1 and SIAs, there was a more rapid decline in measles burden, but large-scale outbreaks were predicted (figure 2). The largest absolute burden reduction attributable to MCV1, MCV2, and SIAs in comparison with no vaccination during 2000–20 was in India, China, and Nigeria (appendix p 11), which are the countries with the highest global IHME measles incidence estimates and largest population sizes of the 14 analysed countries.

Compared with no vaccination, we estimated that MCV1 alone prevented 824 million measles cases and 9·6 million deaths from measles during 2000–20 in the 14 countries (table 1; appendix p 8). SIAs conducted in these countries were estimated to have prevented a further 256 million cases and 4·2 million deaths compared with MCV1 alone. MCV2, as used by these countries, was estimated to have prevented 108 million cases and 404 000 deaths compared with MCV1 alone. SIAs showed more effects on burden reduction than MCV2 when added to MCV1, as indicated by the increased number of prevented cases and deaths in all countries except China and Ukraine. Furthermore, the strategy of MCV1, MCV2, and SIAs was predicted to have incrementally averted 303 million cases and 4·4 million deaths compared with MCV1 alone.

**Table 1:** Estimated number of prevented cases, in thousands, across different vaccination delivery strategies reported by each country (2000–20)

		**Compared with no vaccination**		**Compared with MCV1 alone**		
		MCV1, MCV2, and SIAs	MCV1 alone	MCV1 and SIAs	MCV1 and MCV2	MCV1, MCV2, and SIAs
MCV2 introduction before 2017						
	India	349 481	295 563	41 272	28 182	53 918
	Indonesia	83 082	61 350	16 176	9902	21 732
	China	321 262	257 140	32 643	57 289	64 122
	Philippines	42 549	29 887	11 448	3164	12 661
	Pakistan	76 409	49 163	26 681	7070	27 246
	Angola	15 515	6780	8651	433	8735
	Ukraine	7306	6090	102	1132	1217
	Malawi	9944	7003	2926	313	2941
MCV2 introduction during 2017–20						
	Nigeria	93 009	39 343	53 667	251	53 667
	Ethiopia	49 569	23 045	26 357	484	26 525
	Madagascar	12 033	6935	5087	25	5098
No MCV2 introduction until 2020						
	Uganda	23 922	15 024	8898	..	8898
	DR Congo	40 965	23 429	17 537	..	17537
	Somalia	7634	2931	4703	..	4703
Total		1 132 682	823 682	256 146	108 245	309 000

Five pairs of strategies were compared with two comparator strategies to assess the health effects of additional vaccination delivery strategies as reported by each country. MCV2 effects begin in the year when WUENIC first reports MCV2 coverage (appendix p 1). Note that the strategy of MCV1, MCV2, and SIAs compared with no vaccination (represented in the first column of data) does not depict an actual historical policy implementation for vaccination strategies. Countries are presented in order of their introduction year of MCV2 and magnitude of measles burden (appendix p 1). Sums of the prevented cases in the 14 countries are presented in the last row of the table. Entries with no value correspond to options involving MCV2 in the three countries that have not yet introduced MCV2 (ie, Uganda, DR Congo, and Somalia), so prevented cases cannot be estimated. MCV1=the first routine dose of measles-containing vaccine. MCV2=the second routine dose of measles-containing vaccine. SIA=supplementary immunisation activity. WUENIC=WHO and UNICEF estimates of national immunisation coverage.

Compared with no vaccination, incremental NNVs for MCV1 ranged between 1·27 and 1·46, with a median NNV of 1·41 (IQR 1·35–1·44) across the 14 analysed countries. In comparison with MCV1 alone, SIAs had a median NNV of 6·02 (5·30–7·68), which was greater than including MCV2 in seven of the 11 countries that have introduced MCV2 (median NNV 5·41, 4·76–6·11; table 2). The opposite trend of incremental NNV was observed in Nigeria, Ethiopia, Angola, and Madagascar, where frequent SIAs took place and MCV2 had only been introduced in 2015 at the earliest (appendix p 1). Furthermore, including SIAs when both MCV1 and MCV2 were used led to a median NNV of 6·44 (5·36–9·78), whereas including MCV2 when both MCV1 and SIAs were used resulted in a median NNV of 17·0 (9·34–42·33). There is diminishing return in efficiency for including an additional vaccination delivery strategy when multiple strategies are already in use.

**Table 2:** Incremental NNV to prevent a measles case across different vaccination delivery strategies (2000–20)

	**MCV1 *vs* no vaccination**	**MCV1 and SIAs *vs* MCV1**	**MCV1 and MCV2 *vs* MCV1**	**MCV1, MCV2, and SIAs *vs* MCV1 and SIAs**	**MCV1, MCV2, and SIAs *vs* MCV1 and MCV2**
**MCV2 introduction before 2017**					
India	1·35	10·20	5·41	11·57	16·13
Indonesia	1·27	7·54	4·73	7·75	10·00
China	1·34	8·23	4·59	7·57	35·73
Philippines	1·33	5·57	4·39	10·92	6·65
Pakistan	1·44	6·95	4·80	57·52	9·12
Angola	1·45	4·48	5·32	27·14	4·66
Ukraine	1·26	12·51	6·10	6·17	14·72
Malawi	1·36	7·72	6·59	132·46	8·55
**MCV2 introduction during 2017–20**					
Nigeria	1·46	4·53	5·78	918 368·12	4·55
Ethiopia	1·42	5·21	6·12	17·03	5·27
Madagascar	1·40	3·67	8·16	17·84	3·68
**No MCV2 introduction until 2020**					
Uganda	1·41	6·22	..	..	6·22
DR Congo	1·44	5·61	..	..	5·61
Somalia	1·45	5·83	..	..	5·83
Median	1·40 (1·35–1·44)	6·02 (5·30–7·68)	5·41 (4·76–6·11)	17·03 (9.34–42.33)	6·44 (5·36–9·78)

Data are NNV. Incremental NNV is defined as the ratio of additional doses given to incremental prevented cases in a vaccine delivery strategy compared with its comparator. The median NNVs among countries with applicable values for the five comparison pairs are presented in the last row of the table. Entries with no NNV value correspond to options involving MCV2 in the three countries that have not yet introduced MCV2 (ie, Uganda, DR Congo, and Somalia), so NNV cannot be estimated. As MCV2 did not contribute to burden reduction in these three countries, the incremental NNV values are the same between the MCV1, MCV2, and SIAs strategy *vs* the MCV1 and MCV2 strategy, and the MCV1 and SIAs strategy *vs* the MCV1 strategy. For the MCV1, MCV2, and SIAs strategy *vs* the MCV1 and SIAs strategy, the incremental NNV in Nigeria is exceptionally large due to a small number of prevented cases from MCV2 introduction in 2019. MCV1=the first routine dose of measles-containing vaccine. MCV2=the second routine dose of measles-containing vaccine. NNV=number needed to vaccinate to prevent a case. SIA=supplementary immunisation activity.

**Figure 3:**
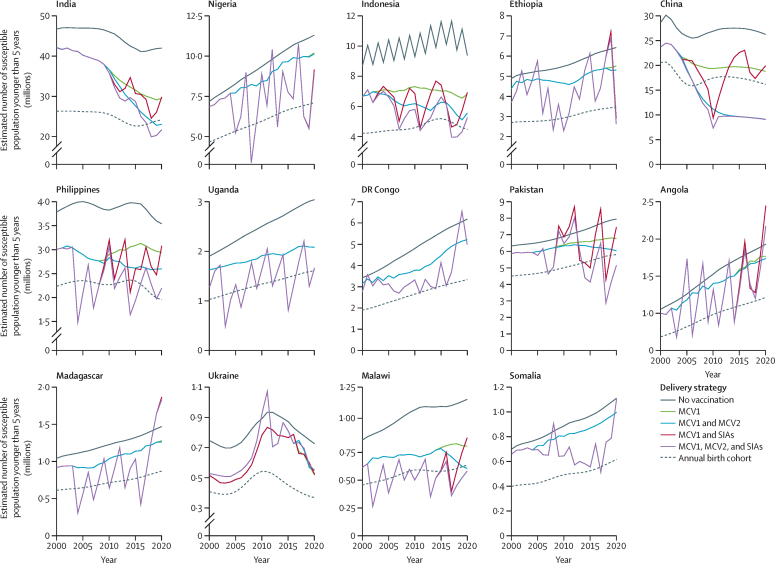
Susceptible population younger than 5 years by vaccination delivery strategy (2000–20) Estimated total numbers of susceptible people younger than 5 years under different vaccination delivery strategies compared with the size of birth cohort. For countries that have not yet introduced MCV2 (ie, Uganda, DR Congo, and Somalia), there are overlapping trends for incidence rates for the delivery strategies of MCV1 and MCV2 (blue lines) and MCV1 only (green lines) and the delivery strategies of MCV1, MCV2, and SIAs (purple lines) and MCV1 and SIAs (red lines). Overlapping trends are also seen in most analysed years in countries that introduced MCV2 in 2017 or later (ie, Nigeria, Ethiopia, and Madagascar). In Indonesia, the fluctuations seen in the no vaccination strategy are the result of dynamic sizes of the susceptible population affected by natural seasonality of measles transmission. MCV1=the first routine dose of measles-containing vaccine. MCV2=the second routine dose of measles-containing vaccine. SIA=supplementary immunisation activity.

The estimated total number of susceptible children younger than 5 years shows varying patterns by vaccination delivery strategy (figure 3). Historical coverage rates with MCV1 and MCV2 reduced measles susceptibility compared with the counterfactual scenario with no vaccination, but the numbers of susceptible children remained higher than one birth cohort in 11 (73%) of the analysed countries by 2020. China was an exception, where high MCV1 and MCV2 coverage successfully kept the susceptible population under the threshold of one birth cohort since 2007. Furthermore, sustained high MCV2 coverage in India resulted in the number of susceptible children being less than one birth cohort from mid-2017 onwards. Despite several rebounds of the susceptible population (ie, when the susceptible population is larger than the size of one birth cohort) during 2000–20, MCV1 and SIAs had more potential to reduce the number of susceptible children than MCV1 and MCV2. However, we estimated that in two countries (ie, India and Ukraine), MCV1 and SIAs would not have reduced the number of susceptible children to below the birth cohort in any year (appendix p 7). Overall, measles vaccination strategies as reported by these countries were estimated to have reduced the number of susceptible children below the birth cohort in a median 24% (14–37) of years between 2000 and 2020.

**Figure 4:**
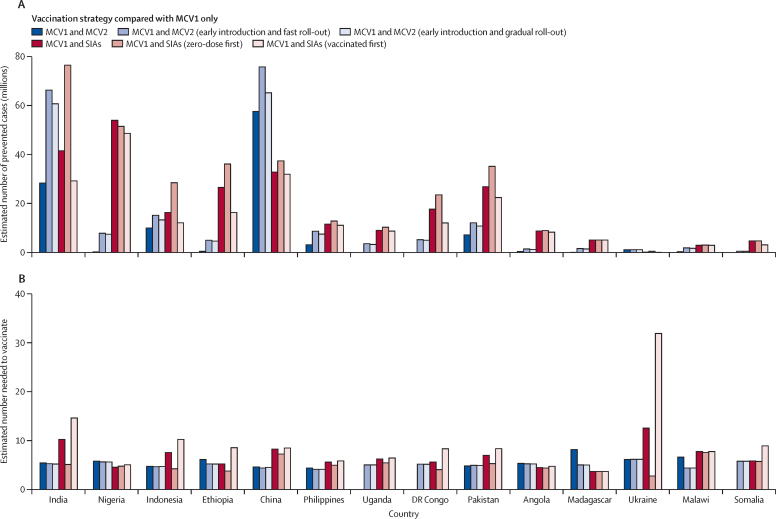
Prevented cases and number needed to vaccinate to prevent a measles case under alternative assumptions for early MCV2 introduction and different SIA dose distribution (A) Prevented cases. (B) Number needed to vaccinate to prevent a measles case. In the sensitivity analysis, we modelled the incremental effect and efficiency of vaccination under the alternative assumptions of MCV2 introduction and SIA distribution. The incremental effects of each of the strategies were compared with the strategy in which MCV1 was already in use. The incremental effects of each of the strategies are compared with the strategy in which MCV1 was already in use. In the main analysis, MCV2 was introduced on the basis of its historical WUENIC coverage (dark blue) and SIAs were distributed with an assumption that 7·7% of children were less likely to be reached by vaccination than the rest of the targeted population (red). The alternative MCV2 assumption indicates early introduction of MCV2 in 2000 with coverage inputs from the appendix (p 9; light blue). Three countries that have not yet introduced MCV2 (ie, Uganda, DR Congo, and Somalia) have missing estimates for the original strategy with MCV1 and MCV2. Two alternative assumptions for SIA distribution were evaluated, including prioritisation of children who had not received any MCV doses (pink) and prioritising children who had been previously vaccinated (dark red). MCV=measles-containing vaccine. MCV1=the first routine dose of measles-containing vaccine. MCV2=the second routine dose of measles-containing vaccine. SIA=supplementary immunisation activity. WUENIC=WHO and UNICEF Estimates of National Immunization Coverage.

We estimated prevented cases and incremental NNVs under alternative assumptions for MCV2 early introduction and SIA dose distribution (figure 4; appendix pp 9–10). Compared with MCV1 alone, early introduction of MCV2, either under fast or gradual roll-out, would have prevented more cases than occurred when MCV2 was actually introduced, resulting in a further estimated reduction of 75–97 million measles cases across the 14 analysed countries. Only a slight improvement in efficiency was seen from early MCV2 introduction, with a median NNV reducing from 5·41 (4·76–6·11) to 5·09 (4·71–5·25). The distribution of SIA doses between zero-dose and already-vaccinated populations had a strong effect on the incremental effects and efficiency of vaccination. When MCV1 was already in use, successfully directing SIA doses first to children in the zero-dose population then to children who were already vaccinated was estimated to prevent more cases of measles than early MCV2 introduction in all countries except China. This observation was particularly apparent when MCV1 coverage was low, so there was a greater proportion of children in the zero-dose population who were eligible for vaccination with an additional dose via an SIA. Prioritising the zero-dose population for SIA doses was estimated to improve efficiency (median NNV 4·84 [4·09–5·40] *vs* 6·02 [5·30–7·68] in the main analysis) for countries with low routine-immunisation coverage, such as Nigeria and DR Congo. Conversely, when SIA doses first reached children who were already vaccinated, the median NNV increased to 8·32 (5·97–8·81) and was estimated to substantially reduce the number of prevented cases of measles compared with the main analysis.

## Discussion

Using MCV coverage data from 2000 to 2020, we investigated and estimated the relative effects and efficiency of different MCV strategies in 14 countries with high measles burden that include a wide range of socioeconomic, demographic, and immunisation-service settings.

The use of MCV1 resulted in the highest relative health effects of any dose and the best efficiency in reducing measles burden. The strategy of MCV1 and SIAs can more effectively keep the susceptible-population size less than the size of one birth cohort and had a bigger effect on predicted measles incidence than MCV1 and MCV2 together, when both strategies were compared with MCV1 alone. Overall, SIAs reduced the susceptible-population size more than MCV1 and MCV2, whereas the efficiency of SIAs, as assessed by NNV, to prevent a measles case was lower than the efficiency of MCV2. However, there was variation between countries in the relative efficiency of each incremental strategy. The strategies used between 2000 and 2020 in the 14 included countries substantially reduced measles burden compared with a no-vaccination strategy but, other than in China, were not predicted to prevent large outbreaks. This finding is consistent with other analyses in low-income and middle-income countries.^30^

The high effects but reduced efficiency of SIAs could also be interpreted from the viewpoint of dose delivery—although SIAs could be delivered to more people than MCV1 and MCV2, many doses were predicted to reach children who had previously been vaccinated (appendix p 12). Repeated vaccinations were seen more often in countries with high routine immunisation coverage than in countries with low routine immunisation coverage, such as Madagascar, where SIAs remained an important strategy to reach children who were unvaccinated. Compared with SIAs, MCV2 showed relatively less effect in countries with low MCV2 coverage such as DR Congo, Nigeria, Angola, Ethiopia, and Somalia, even under the assumption of early MCV2 introduction, compared with countries with higher sustained MCV1 coverage such as China and Malawi. Accompanied by better reach of SIAs to children in the zero-dose population, early introduction of MCV2 could have substantially reduced incidence over time. In countries that implemented routine MCV2 early and maintained high levels of coverage, such as China and India, there was little difference in estimated measles incidence rate between historical strategies and optimal assumptions for MCV delivery when MCV2 was introduced in 2000 and SIA doses were given first to children in the zero-dose population (appendix p 13). This finding suggests that, in the future, SIAs might be needed less often if high coverage of MCV2 can be successfully attained, maintained, and aligned with WHO recommendations.^8^ These results might also be generalisable to other vaccine preventable diseases; for example, similar findings have been shown for polio, such that if high baseline routine immunisation can be maintained, SIA frequency can be reduced with low probability of an outbreak.^31^

WHO advises countries that have not yet introduced rubella-containing vaccine (RCV) to do so via nationwide, non-selective SIAs of measles–rubella vaccine until at least age 15 years. Once RCV has been introduced, the timing and extent of further SIAs depends on the epidemiology of measles, which has higher transmissibility than rubella. The Measles and Rubella Strategic Framework 2021–2030 emphasises shifting from a so-called one-size-fits-all approach to focus on effective local approaches for vaccinating hard-to-reach populations with MCV.^32^ For high-burden countries to achieve high levels of coverage and meet targets for measles elimination, SIAs should be strengthened but could be made more efficient and designed to fit local demand. If SIA efficiency is low in a particular setting, as shown by a high predicted NNV to prevent a case, but SIAs consistently result in a greater burden reduction than MCV2, investing in mechanisms to improve efficiency through improved surveillance and coverage data to target SIAs and improving so-called mop-up activities in specific areas where the virus is known or suspected to be circulating immediately after a campaign^33^ will be valuable. Mop-up activities involve going to areas where the reported number of doses administered in the SIA was lower than the target population, or to places where a rapid-coverage evaluation shows low coverage, and conducting special vaccination activities to increase coverage (eg, going to each house to identify and vaccinate any children who have not been reached).

We did not explore potential differences in effectiveness and efficiency between selective and non-selective approaches.^34^ Some countries have implemented selective SIAs, but further empirical data are needed on the feasibility of this approach in a range of contexts. Further studies should also assess the combined effectiveness and efficiency of integrated campaigns,^35^ which deliver multiple vaccines or include other interventions, such as nutritional screening.

Our study has limitations. First, SIA doses were assumed to be randomly delivered to their target population, except for a fixed proportion of children who were assumed to be less likely to be reached by childhood immunisation programmes.^24^ The extent to which in-practice doses are correlated with, or independent of, previous vaccination status is unknown because only a minority of countries report high-quality, post-campaign-coverage surveys to WHO and even fewer surveys report SIA coverage and previous measles vaccination status.^36^ Other household surveys, such as Demographic and Health Surveys, try to capture specific information on measles vaccination,^37^ but the ability to compare SIA dose receipt among children in the zero-dose population or children who were previously vaccinated is constrained by the low proportion of children with documentation of routine vaccination and potential misclassification of routine or SIA vaccination when relying on parental recall.^37^ For each measles vaccination campaign, however, the size of zero-dose population reached by SIAs varies depending on local routine coverage and SIA-implementation approach.^36^ Furthermore, SIA coverage reported in the WHO record might be overestimated,^36^, ^38^, ^39^ possibly due to vaccinating non-target populations or not capturing unreached populations in the denominator.

Second, MCV2 effect based on historical coverage could be underestimated, given our purposeful selection of high-burden countries that mostly had low coverage. Moreover, differences exist between MCV2 recommended policies and vaccination in practice. For example, lessons learned from MCV2 routine immunisation introduction in Africa found that, in practice, to reduce vaccine wastage, vaccinations were only administered on days when 10 or more children were present, missing opportunities for vaccination.^40^ Furthermore, the interplay between MCV1, MCV2, and SIAs should be considered when planning future measles vaccination strategies.

Third, although DynaMICE is a dynamic transmission model that captures the indirect effect (eg, herd immunity) of vaccination, it does not capture international case importation. Furthermore, due to model limitations in simulating measles outbreaks in subnational areas, differentiation between outbreak-response SIAs and preventive SIAs was not explored in our analysis. Additionally, accurately accounting for the effect of subnational SIAs and subnational variations in both routine immunisation and SIA coverage remains a challenge as subnational data on SIAs are not regularly collected. The potential effects of subnational variation in key determinants of measles transmission, such as birth rates, routine and SIA vaccination coverage, and migration, were also not assessed.

Finally, NNV is not applicable for comparison between strategies when an alternative strategy does not prevent additional cases and there is no established threshold to establish whether the efficiency of an immunisation programme is acceptable.^41^ Further data, such as the costs of vaccine procurement and delivery, will be useful in understanding the cost-effectiveness of immunisation programmes.

The resources required for SIAs, including economic and human resources and logistical challenges, can be major deterrents to their implementation. Despite several unknowns regarding interpretation of estimated NNVs, our results show that routine MCV2 is not always more efficient than SIAs. Furthermore, the current trend towards including multiple interventions in a single SIA or integrating many of the components of SIA planning across different interventions might increase efficiency, although monitoring the effectiveness of integrated campaigns will be important.^42^, ^43^ There is a need to improve the evaluation of SIAs to identify how they could increase efficiency, transfer best practices between countries, and ensure adequate and timely funding for SIA implementation and evaluation.

We assessed the incremental effects and efficiency of different measles vaccination strategies to inform future decisions about vaccination planning and policies. Understanding the relative effects and efficiency of the first routine dose, the second routine dose, and SIAs of MCV will assist stakeholders in assessing the value of measles vaccination programmes and further identify improved pathways towards measles elimination.

## Data sharing

The coverage data and simulation code used for analysis can be accessed through GitHub (https://github.com/hfu915/dynamice_ph) and are currently available.

## Declaration of interests

We declare no competing interests.
