# Supplementary material for: Health effects of routine measles vaccination and supplementary immunisation activities in 14 high-burden countries: a Dynamic Measles Immunization Calculation Engine (DynaMICE) modelling study
Source: Lancet Glob Health. 2023 Jul 18;11(8):e1194–204. doi: 10.1016/S2214-109X(23)00220-6 (PMC10369016; doi:10.1016/S2214-109X(23)00220-6)
Supplement: Supplementary appendix [file mmc1.pdf]

THE LANCET
Global Health

Supplementary appendix

This appendix formed part of the original submission and has been peer reviewed. We post it as supplied by the authors.

Supplement to: Auzenbergs M, Fu H, Abbas K, Procter SR, Cutts FT, Jit M. Health effects of routine measles vaccination and supplementary immunisation activities in 14 high-burden countries: a Dynamic Measles Immunization Calculation Engine (DynaMICE) modelling study. *Lancet Glob Health* 2023; **11:** e1194–204.

Appendix

Health effects of routine measles vaccination and supplementary immunisation activities in 14 high-burden countries: a Dynamic Measles Immunization Calculation Engine (DynaMICE) modelling study

Table of Contents

Table S1……………………………………………………………………………………………1
Table S2……………………………………………………………………………………………2
Table S3……………………………………………………………………………………………7
Table S4……………………………………………………………………………………………8
Figure S1……………………………………………………………………………………………9
Figure S2……………………………………………………………………………………………10
Figure S3……………………………………………………………………………………………11
Figure S4……………………………………………………………………………………………12
Figure S5……………………………………………………………………………………………13
Figure S6……………………………………………………………………………………………14
Figure S7……………………………………………………………………………………………15
Figure S8……………………………………………………………………………………………16

Table S1. Number of cases across modelled countries 2010-2019 and vaccination schedules

Country	Reported number of cases (WUENIC)	Estimated number of cases (IHME)	Nationally recommended age for MCV1	Nationally recommended age for MCV2	Year of MCV2 introduction
India	192566	112263832	9—12 months	16—24 months	2011
Nigeria	169394	15381229	9 months	15 months	2019
Indonesia	115974	15373892	9 months	18 months	2003
Ethiopia	59824	10108569	9 months	15 months	2019
China	213832	6171658	8 months	18 months	2005
Philippines	149325	6050672	9 months	12—15 months	2009
Uganda	20029	4555096	9 months	-	-
DRC	791259	3976920	9 months	-	-
Pakistan	74209	3271893	9 months	15 months	2009
Angola	30564	3246826	9 months	15 months	2015
Madagascar	234682	2966145	9 months	15—18 months	2020
Ukraine	129608	154625	12 months	6 years	2000
Malawi	118775	852873	9 months	15 months	2015
Somalia	80769	2440208	9 months	-	-

Table S1. For countries without available data on the recommended MCV2 schedules, we assumed the age at vaccination to be 15–18 months old [8]. In China and Philippines, where multiple types of vaccines are in use, we adopted the vaccination schedules from the two doses of MMR (measles-mumps-rubella). Year of MCV2 introduction was assumed to be the first year a country has available coverage data in the WUENIC database. (-) indicates a country has not yet introduced MCV2 and therefore, does not have a recommended age for MCV2 vaccination. In the model, MCV1 is assumed to deliver to unvaccinated infants at 9 months old in most countries, and MCV2 to previously vaccinated children at the recommended age of each country. IHME: Institute for Health Metrics and Evaluation. MCV1: the first routine dose of measles-containing vaccine. MCV2: the second routine dose of measles-containing vaccine. WUENIC: WHO and UNICEF Estimates of National Immunization Coverage.

Table S2. Supplementary immunisation activities in 14 countries, 2000-2020.

Year	Month	Target age group	Target population size	Reached doses	Coverage - target	Coverage - country-level
**India**						
2000	-	9-59m	974034	739417	75.9%	0.7%
2001	-	9-59m	1384891	962474	69.5%	0.9%
2010	Dec	9m-10y	10469901	9367822	89.5%	3.6%
2011	Apr	9m-10y	3375785	2709014	80.2%	1.0%
2011	Dec	9m-10y	30751228	27919442	90.8%	10.7%
2012	Mar	9m-9y	9416352	8215227	87.2%	3.5%
2012	Dec	9m-9y	40717834	36976587	90.8%	15.7%
2013	Jun	9m-9y	36012805	33639706	93.4%	14.4%
2017	Feb	9m-15y	16033000	15845000	98.8%	4.2%
2017	Feb	9m-15y	320000	312000	97.5%	0.1%
2017	Apr	9m-15y	16000	12000	75.0%	0.0%
2017	Apr	9m-15y	17605000	16953000	96.3%	4.5%
2017	Apr	9m-15y	304000	266000	87.5%	0.1%
2017	Aug	9m-15y	11854000	11458000	96.7%	3.0%
2017	Aug	9m-15y	114000	115000	100.9%	0.0%
2017	Aug	9m-15y	58000	62000	106.9%	0.0%
2017	Sep	9m-15y	9001000	9148000	101.6%	2.4%
2017	Sep	9m-15y	310000	301000	97.1%	0.1%
2017	Sep	9m-15y	1774000	1808000	101.9%	0.5%
2017	Dec	9m-15y	2836000	2876000	101.4%	0.8%
2017	Dec	9m-15y	7655000	6488000	84.8%	1.7%
2018	Feb	9m-15y	438000	443000	101.1%	0.1%
2018	Mar	9m-15y	11225000	11037000	98.3%	3.0%
2018	May	9m-15y	324000	324000	100.0%	0.1%
2018	May	9m-15y	818000	793000	96.9%	0.2%
2018	Jun	9m-15y	83000	78000	94.0%	0.0%
2018	Jul	9m-15y	6964000	6684000	96.0%	1.8%
2018	Jul	9m-15y	7438000	7364000	99.0%	2.0%
2018	Sep	9m-15y	15157000	14560000	96.1%	3.9%
2018	Oct	9m-15y	10602000	10257000	96.7%	2.7%
2018	Oct	9m-15y	1156000	1107000	95.8%	0.3%
2018	Oct	9m-15y	9209000	9028000	98.0%	2.4%
2018	Nov	9m-15y	7778000	7792000	100.2%	2.1%
2018	Nov	9m-15y	3762000	3757000	99.9%	1.0%
2018	Nov	9m-15y	449000	439000	97.8%	0.1%
2018	Nov	9m-15y	956000	897000	93.8%	0.2%
2018	Dec	9m-15y	76403000	75720000	99.1%	20.3%
2018	Dec	9m-15y	29052000	25866000	89.0%	6.9%
2019	Feb	9m-15y	37757000	38415000	101.7%	10.4%
2019	Feb	9m-15y	23245000	22822000	98.2%	6.1%
**Nigeria**						
2005	Dec	9m-15y	29500000	28538974	96.7%	47.8%
2006	Oct	9m-15y	31630011	26353793	83.3%	42.9%
2007	Jan	9-59m	2583480	2308527	89.4%	10.7%
2007	Mar	6y-17y	662164	517410	78.1%	1.2%
2008	Dec	9-59m	29828229	28848102	96.7%	130.4%
2011	Feb	9-59m	28272893	28435589	100.6%	119.3%
2013	Oct	9-59m	15957208	17004058	106.6%	68.0%
2013	Nov	9-59m	13344281	13575608	101.7%	54.3%
2015	Nov	6m-10y	23967617	24069024	100.4%	40.8%
2016	Jan	9-59m	14577013	19065787	130.8%	71.2%
2018	Feb	9-59m	37412277	40044875	107.0%	144.4%
2018	Mar	9-59m	15969475	16955354	106.2%	61.1%
2019	Nov	9-59m	20531083	21417932	104.3%	76.1%

**Indonesia**						
2000	-	6-12y	6665950	6341407	95.1%	20.8%
2000	-	6-59m	1142183	948012	83.0%	4.9%
2002	Sep	0-59m	166087	155101	93.4%	0.8%
2002	Nov	9-59m	2667343	2031029	76.1%	11.0%
2003	Oct	6-12y	1030445	980754	95.2%	3.1%
2004	-	6-12y	2180918	2062556	94.6%	6.5%
2005	Feb	6m-15y	4836094	4642650	96.0%	6.6%
2005	-	6m-15y	679230	615577	90.6%	0.9%
2006	Jan	6-12y	3161323	3049844	96.5%	9.6%
2006	May	6m-5y	234528	220777	94.1%	0.9%
2006	Aug	6m-5y	3743568	3440698	91.9%	13.9%
2007	Feb	6-59m	11237274	10099534	89.9%	49.7%
2007	Feb	6m-12y	2692912	2863068	106.3%	5.0%
2007	Aug	6y-12y	2569350	2609301	101.6%	8.2%
2007	Aug	6-59m	3679318	3499242	95.1%	17.2%
2008	Oct	1-3y	11203	8730	77.9%	0.1%
2009	Oct	9-59m	141685	126800	89.5%	0.6%
2009	Oct	9-59m	219765	126699	57.7%	0.6%
2009	Oct	9-59m	1763122	1700834	96.5%	8.7%
2010	Oct	9-59m	3619024	3294315	91.0%	16.7%
2011	Nov	9-59m	11989559	11365665	94.8%	57.8%
2016	Aug	9-59m	4222172	3638183	86.2%	17.3%
2017	Aug	9m-15y	34964384	35307148	101.0%	48.9%
2018	Oct	9m-15y	31963154	23453882	73.4%	32.5%
**Ethiopia**						
2000	Jul	9-59m	3800000	3610000	95.0%	35.0%
2001	Jan	9-59m	3026147	2346464	77.5%	21.9%
2001	Dec	9-59m	2166232	1646336	76.0%	15.4%
2002	Nov	9m-14y	2316214	2277988	98.3%	7.4%
2003	Aug	6m-14y	5605502	5101007	91.0%	15.8%
2004	Apr	6m-14y	8835802	7422074	84.0%	22.4%
2005	-	6m-14y	198456	136935	69.0%	0.4%
2005	Sep	9-59m	1073066	987221	92.0%	8.6%
2006	Apr	9-59m	11688720	10169187	87.0%	87.9%
2007	Nov	6-59m	1117345	1072701	96.0%	8.7%
2008	Nov	6-59m	11791819	10848474	92.0%	86.9%
2009	Jan	6-59m	773910	662168	85.6%	5.2%
2009	Jan	6-59m	279102	264134	94.6%	2.1%
2009	Jan	6-59m	62504	57762	92.4%	0.5%
2009	Jun	6-59m	285644	266621	93.3%	2.1%
2010	Mar	6-59m	1057327	961798	91.0%	7.5%
2010	Oct	9-47m	7656367	8171534	106.7%	88.1%
2011	Feb	9-47m	774658	757421	97.8%	8.1%
2011	Oct	6m-14y	7326463	7034264	96.0%	18.3%
2013	Jun	9-59m	11873928	11609484	97.8%	92.3%
2016	Apr	6m-<15y	25706550	24986589	97.2%	60.1%
2017	Mar	9m-14y	22035787	21225199	96.3%	51.3%
2017	Aug	6-179m	2579178	2524841	97.9%	6.0%
2020	Jul	9-59m	14135353	13970822	98.8%	98.5%
**China**						
2003	Nov	8m-12y	831600	819732	98.6%	0.4%
2004	Nov	8m-12y	10000000	7791796	77.9%	3.5%
2005	Mar	8m-14y	4222349	4032343	95.5%	1.5%
2005	May	8m-25y	2410000	2289500	95.0%	0.4%
2005	Jul	1-14y	5193749	4300424	82.8%	1.7%
2005	Sep	8m-14y	8000000	4800000	60.0%	1.8%
2006	Mar	8m-14y	6200000	5900000	95.2%	2.3%

2007	Sep	8m-14y	20400000	20100000	98.5%	8.1%
2008	Jul	8m-14y	35705379	34983574	98.0%	14.2%
2008	Nov	8m-6y	11842830	11570750	97.7%	11.0%
2009	Apr	8m-14y	5114101	5008565	97.9%	2.0%
2009	Jul	8m-14y	96487581	94167415	97.6%	38.4%
2010	Sep	8m-4y	106060935	102300000	96.5%	140.1%
**Philippines**						
2002	Jun	9-59m	507463	500897	98.7%	5.3%
2004	Mar	9m-7y	18394880	17291555	94.0%	108.0%
2007	Nov	9-48m	8648864	8201862	94.8%	85.7%
2009	Aug	15-23m	787693	459682	58.4%	7.8%
2010	Jul	9-59m	-	420129	-	4.5%
2011	May	9-95m	18651791	15649907	83.9%	98.8%
2013	Nov	6-59m	-	1937471	-	19.0%
2013	Dec	6-59m	-	108783	-	1.1%
2014	Jan	6-36m	2183971	1695930	77.7%	29.3%
2014	Sep	9-59m	11485540	10402489	90.6%	107.3%
2018	May	6-59m	4205517	2893466	68.8%	28.9%
2018	Oct	6-59m	6604059	2089432	31.6%	20.9%
2019	Apr	6-59m	3784099	3920103	103.6%	40.1%
2019	Jul	5-12y	8575452	2457514	28.7%	13.7%
2019	Jul	13y-100y	2179336	947677	43.5%	1.2%
**Uganda**						
2000	Mar	9-59m	1596240	1218737	76.4%	30.7%
2000	Nov	9-59m	1030490	957876	93.0%	24.2%
2001	Oct	9m-14y	503904	614156	121.9%	5.4%
2003	Oct	6m-14y	12861020	13457127	104.6%	108.8%
2005	Feb	9-23m	389696	557869	143.2%	41.5%
2006	Aug	6-59m	5263090	5239221	99.5%	103.9%
2009	Jun	9-47m	4699748	4893634	104.1%	122.7%
2012	May	6-59m	6314309	6283441	99.5%	107.0%
2015	Oct	6-59m	6658881	6349182	95.3%	101.0%
2019	Oct	9m-<15y	18200969	19432256	106.8%	100.2%
**DRC**						
2000	Oct	9-59m	1926879	1395451	72.4%	19.3%
2003	Jan	6m-15y	5774245	5554824	96.2%	23.5%
2003	Feb	6m-15y	100000	108000	108.0%	0.5%
2004	Oct	6m-15y	10055523	8604754	85.6%	35.3%
2005	Oct	6m-15y	7817076	6957653	89.0%	27.6%
2006	Aug	6-59m	2189069	2158329	98.6%	22.9%
2006	Nov	6m-15y	7164815	6966200	97.2%	26.7%
2007	Aug	6-59m	3736672	3768794	100.9%	38.5%
2008	Nov	6-59m	2852430	2811092	98.6%	27.8%
2009	Nov	6-59m	2593478	2412168	93.0%	23.1%
2010	Jan	6-59m	1226792	1259363	102.7%	11.7%
2011	Feb	6-59m	1673147	1701315	101.7%	15.2%
2011	Mar	6-59m	2672338	2649574	99.1%	23.7%
2011	Apr	6-59m	415909	430571	103.5%	3.8%
2011	May	6-59m	5227315	4864554	93.1%	43.4%
2011	Jun	6-59m	272392	266811	98.0%	2.4%
2012	Jan	6-59m	845002	948237	112.2%	8.2%
2012	Jan	6-59m	314283	303786	96.7%	2.6%
2012	Jan	6-59m	1253230	1239803	98.9%	10.7%
2012	Aug	6-59m	170530	175467	102.9%	1.5%
2013	Sep	6m-9y	6794574	6813783	100.3%	29.9%
2013	Dec	6m-9y	4087697	4213293	103.1%	18.5%
2014	Mar	6m-9y	5346158	5482612	102.6%	23.3%
2014	May	6m-9y	6137295	6211685	101.2%	26.4%

2014	Jun	6m-9y	2883624	2803550	97.2%	11.9%
2014	Jul	6m-9y	1196822	1163251	97.2%	4.9%
2014	Aug	6m-9y	2858403	2878785	100.7%	12.2%
2016	Aug	6-59m	7081288	7212977	101.9%	55.7%
2016	Oct	6-59m	3725998	3708843	99.5%	28.6%
2017	Feb	6-59m	5291916	5466923	103.3%	41.2%
**Pakistan**						
2005	Dec	12-59m	1600000	1232000	77.0%	6.8%
2007	Mar	9m-15y	2571536	2511837	97.7%	3.8%
2007	Jul	9m-13y	1219364	1282232	105.2%	2.2%
2007	Aug	9m-13y	6890603	6906376	100.2%	11.9%
2007	Nov	9m-13y	21262960	20566497	96.7%	35.5%
2008	Mar	9m-13y	34123305	35315375	103.5%	60.3%
2010	Feb	9m-<13y	15209539	13740906	90.3%	24.8%
2010	Sep	6-59m	7359790	6991065	95.0%	32.5%
2010	Oct	6-59m	988224	1007195	101.9%	4.7%
2011	Jan	9-59m	1401269	1299618	92.7%	6.3%
2011	Jan	9-59m	872287	784337	89.9%	3.8%
2011	Jan	9-59m	5143498	5098071	99.1%	24.8%
2011	Feb	9-59m	2028374	1744206	86.0%	8.5%
2011	Jul	9-59m	225947	205551	91.0%	1.0%
2011	Nov	9-59m	570538	547716	96.0%	2.7%
2012	Dec	9m-9y	1918267	1954175	101.9%	4.5%
2014	May	6m-9y	13418263	14026013	104.5%	30.4%
2014	May	6m-9y	9346563	9432492	100.9%	20.4%
2014	Dec	6m-9y	1438492	1439892	100.1%	3.1%
2015	Feb	6m-10y	29670753	30633406	103.2%	59.7%
2015	Feb	6m-10y	240921	227762	94.5%	0.4%
2015	Feb	6m-10y	165355	204308	123.6%	0.4%
2015	Apr	6m-10y	3474044	3512771	101.1%	6.8%
2015	May	6m-10y	414494	413695	99.8%	0.8%
2015	Aug	6m-10y	1607543	1519242	94.5%	3.0%
2017	Aug	9-59m	1355202	1279819	94.4%	5.7%
2018	Oct	9-59m	35199413	37131234	105.5%	161.4%
**Angola**						
2003	May	9m-14y	7642739	7226105	94.5%	90.8%
2006	Jul	9-59m	3218676	3210160	99.7%	100.2%
2009	Jun	9-59m	3430913	3469806	101.1%	96.3%
2011	Sep	9-59m	5472822	4635248	84.7%	119.1%
2014	Sep	6m-9y	7829940	9169335	117.1%	104.8%
2018	Apr	6m-14y	12858213	12001436	93.3%	86.7%
2019	May	6m-4y	108673	127740	117.5%	2.5%
2019	May	6m-4y	84101	110280	131.1%	2.2%
2019	Jun	6m-4y	141802	164410	115.9%	3.2%
**Madagascar**						
2004	Sep	9m-14y	7626090	7546229	99.0%	100.5%
2007	Oct	9-59m	3123163	3053702	97.8%	112.0%
2010	Oct	9-47m	2603510	2415792	92.8%	108.7%
2013	Oct	9-59m	3610265	3316542	91.9%	109.8%
2016	Oct	9-59m	3715133	3547466	95.5%	111.6%
**Ukraine**						
2008	May	15-29y	8000000	113000	1.4%	1.1%
2017	Oct	1-9y	287240	163782	57.0%	3.7%
2017	Oct	6-9y	231102	154430	66.8%	7.7%
2019	Apr	1-18y	506232	387731	76.6%	4.7%
2019	Apr	6-18y	618756	462407	74.7%	7.8%
**Malawi**						
2002	Aug	9-59m	1583664	1906985	120.4%	106.8%

2005	Sep	9-59m	1851176	2137152	115.4%	111.3%
2008	Oct	9-59m	2120557	2087375	98.4%	99.5%
2010	Aug	9m-15y	6370409	6785428	106.5%	101.9%
2013	Nov	6-59m	2297546	2405018	104.7%	97.5%
2015	May	9-59m	436257	453202	103.9%	19.2%
2017	Jun	9m-14y	7991666	8132788	101.8%	109.9%
**Somalia**						
2005	Nov	9m-15y	384725	319321	83.0%	6.5%
2006	May	9m-15y	2266917	2019717	89.1%	39.7%
2007	Jun	9m-15y	3191161	2774178	86.9%	52.9%
2008	Mar	9m-15y	150000	142654	95.1%	2.6%
2008	Dec	9m-15y	238355	138205	58.0%	2.6%
2009	Mar	9-59m	1483134	909687	61.3%	49.0%
2009	Apr	9-59m	1117384	835927	74.8%	45.0%
2009	Aug	9-59m	380911	276994	72.7%	14.9%
2010	Mar	9-59m	1562336	1335892	85.5%	70.4%
2011	Apr	9-59m	-	2924	-	0.1%
2011	Jul	6m-15y	90000	71653	79.6%	1.2%
2011	Jul	9-59m	169414	151279	89.3%	7.8%
2011	Jul	9-59m	380911	323986	85.1%	16.6%
2011	Aug	6m-15y	745234	656226	88.1%	10.9%
2011	Sep	6m-15y	86373	74300	86.0%	1.2%
2011	Oct	6m-14y	672054	626625	93.2%	11.0%
2012	Mar	6-59m	578457	509042	88.0%	24.0%
2012	Mar	6m-4y	1012879	886033	87.5%	41.8%
2012	Oct	9-59m	959424	872230	90.9%	43.8%
2013	Dec	6m-4y	1029870	923580	89.7%	43.0%
2014	Oct	9-59m	1483574	1306426	88.1%	63.6%
2015	Dec	9m-9y	3884554	3518358	90.6%	82.7%
2016	Aug	9-59m	676557	602136	89.0%	28.2%
2017	Feb	6-59m	4700000	4400000	93.6%	189.4%
2017	May	6m-5y	503812	472033	93.7%	16.9%
2018	Jan	6m-10y	4745484	4424261	93.2%	86.5%
2019	Nov	6-59m	1149117	1061064	92.3%	43.3%

Table S2. WHO records for measles SIAs with a confirmed status of implementation between 2000–2020 in the 14 countries were extracted. We excluded SIAs for the purpose of outbreak response, since they were usually driven by emergency events and restricted in a local area. (-) indicates missing information in the records. Year and month denote the mid-point of each campaign and July 1st is assigned for SIAs with missing dates. Two coverage estimates are presented: one is calculated from the number of target population reported in each SIA (sub-national), and the other is based on the country-level population from the World Population Prospects 2019 at the target age group (national). When there is no information on target population in the WHO records, the sub-national coverage estimates are assumed to be the same as national ones in the analysis. DRC: Democratic Republic of the Congo. SIA: supplementary immunisation activity.

Table S3. Percentage of years over 2000–2020 showing a smaller number of susceptible children than the birth cohort size

Country	No vaccination	MCV1	MCV1+ MCV2	MCV1+SIAs	MCV1+MCV2+SIAs
India	0%	0%	9.5%	0%	14%
Nigeria	0%	0%	0%	24%	24%
Indonesia	0%	0%	0%	9.5%	24%
Ethiopia	0%	0%	0%	14%	14%
China	0%	0%	67%	19%	67%
Philippines	0%	0%	0%	24%	38%
Uganda	0%	0%	0%	38%	38%
DRC	0%	0%	0%	14%	14%
Pakistan	0%	0%	0%	19%	33%
Angola	0%	0%	0%	24%	24%
Madagascar	0%	0%	0%	29%	29%
Ukraine	0%	0%	0%	0%	0%
Malawi	0%	0%	4.8%	48%	57%
Somalia	0%	0%	0%	4.8%	4.8%
Median (25th—75th percentiles)	0% (0%—0%)	0% (0%—0%)	0% (0%—0%)	19% (11%—24%)	24% (14%—37%)

Table S3. This table presents the percentage of years over the analysis period that had an outbreak potential among different vaccination strategies. The outbreak potential in a year is indicated by a larger size of susceptible population under 5 years old compared to the size of birth cohort in each country. The median and 25th and 75th percentiles of percentage among 14 countries are shown in the bottom row. DRC: Democratic Republic of the Congo. MCV1: the first routine dose of measles-containing vaccine. MCV2: the second routine dose of measles-containing vaccine. SIA: supplementary immunisation activity.

Table S4. Averted deaths (thousands) across different vaccination strategies from 2000–2020

Country by MCV2 introduction year	Comparator: no vaccination		Comparator: MCV1 alone		
	MCV1 + MCV2 + SIAs	MCV1 alone	MCV1 + SIAs	MCV1 + MCV2	MCV1 + MCV2 + SIAs
**MCV2 < 2017**					
India	2392	2223	125	84	169
Indonesia	373	308	48	30	65
China	1544	1377	91	149	168
Philippines	199	160	35	9.82	38
Pakistan	1368	989	365	94	379
Angola	531	262	266	9.93	269
Ukraine	56	50	0.18	5.84	6.01
Malawi	337	264	72	5.69	73
**MCV2 > 2017**					
Nigeria	2918	1475	1443	5.19	1443
Ethiopia	1712	858	852	9.45	854
Madagascar	192	135	57	0.21	57
**No MCV2**					
Uganda	798	546	252	0	252
DRC	1352	841	511	0	511
Somalia	229	110	119	0	119
**Total**	14002	9598	4237	404	4403

Table S4. This table presents the total deaths averted in the 5 pairs of vaccination delivery strategies for comparison. Sums of the averted deaths in the 14 countries are shown in the last row of the table. DRC: Democratic Republic of the Congo. MCV1: the first routine dose of measles-containing vaccine. MCV2: the second routine dose of measles-containing vaccine. SIA: supplementary immunisation activity

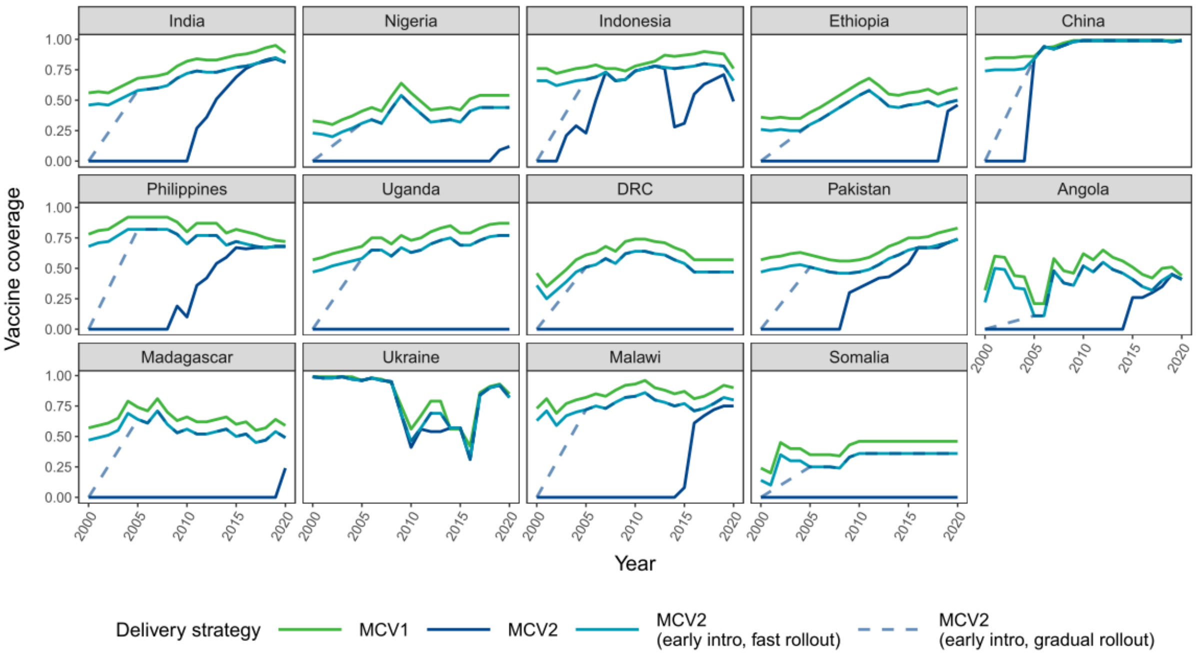

Figure S1. Immunisation coverage for early introduction of MCV2 over 2000–2020.
In the sensitivity analysis, all countries were assumed to introduce MCV2 early in 2000, with each year's coverage 10% lower than MCV1 coverage, or the same as the country's actual MCV2 coverage in that year, whichever was larger. Under fast rollout, MCV2 coverage would reach the assumed level in 2000, whereas MCV2 coverage would increase linearly over 2000–2005. DRC: Democratic Republic of the Congo. MCV1: the first routine dose of measles-containing vaccine. MCV2: the second routine dose of measles-containing vaccine.

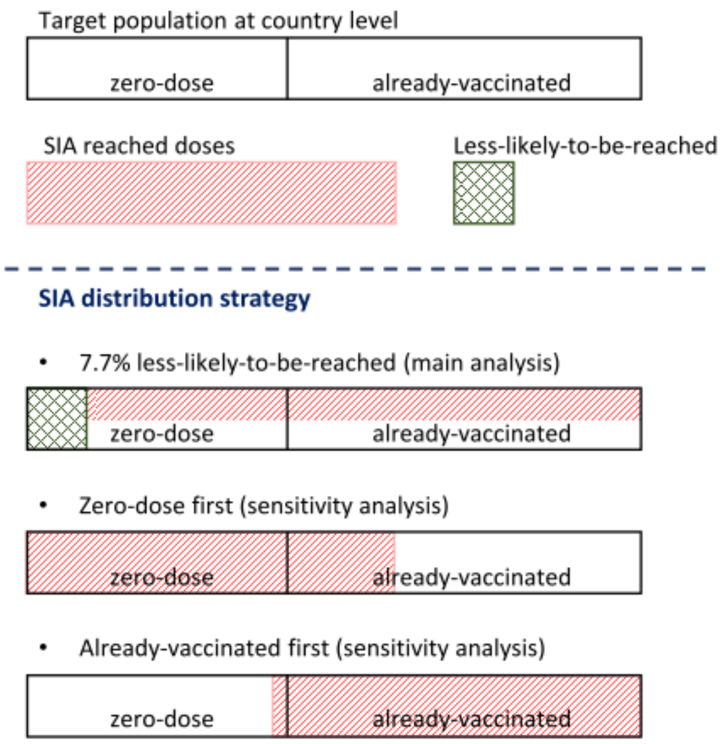

Figure S2. Distribution strategies of SIAs included in the analysis.
In the main analysis, 7.7% of target population are assumed less likely to receive MCV doses through current immunisation programmes and SIAs doses are given randomly to the rest of population. In addition, the distribution strategies to direct SIA doses first to MCV zero-dose and already-vaccinated children are assessed respectively in the sensitivity analysis. MCV: measles-containing vaccine. SIA: supplementary immunisation activity.

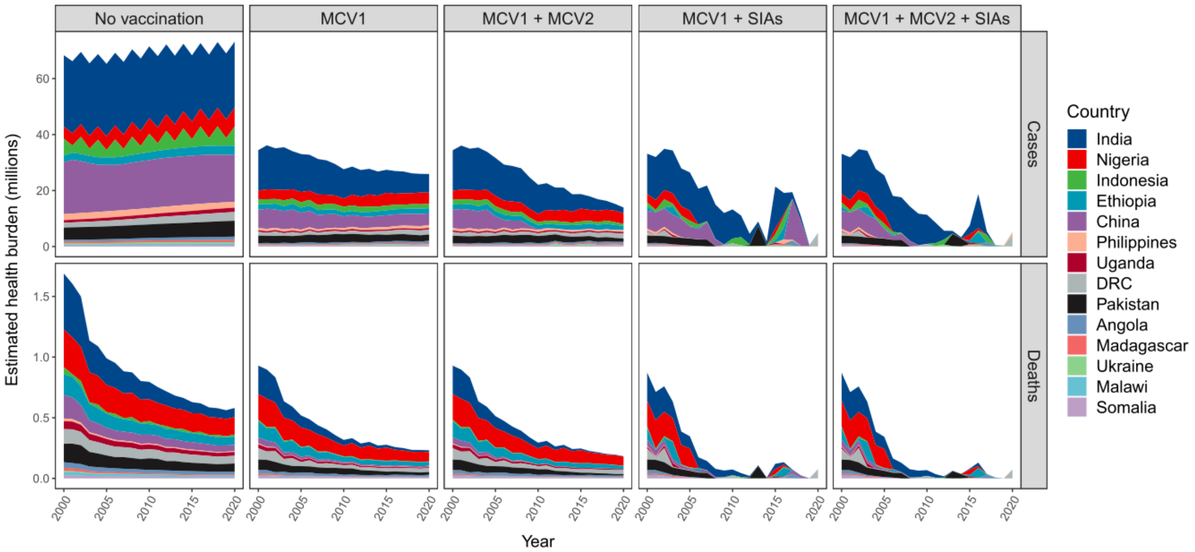

Figure S3. Annual number of measles cases (top row) and deaths (bottom row) across different vaccination delivery strategies over 2000–2020.

Country measles burden is present in different colours and stacked over time. The measles burden decreases with adding vaccination delivery strategies. DRC: Democratic Republic of the Congo. MCV1: the first routine dose of measles-containing vaccine. MCV2: the second routine dose of measles-containing vaccine. SIA: supplementary immunisation activity.

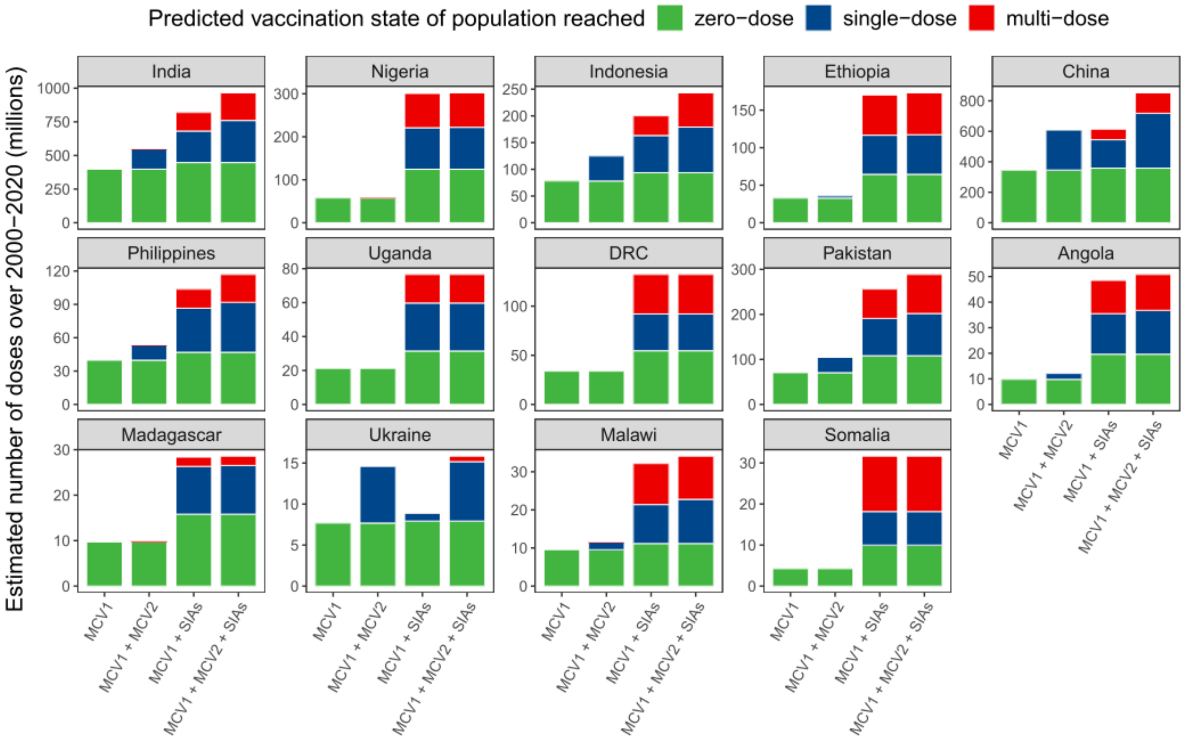

Figure S4. Doses reaching zero-dose, single-dose, and multiple-dose children across different vaccination delivery strategies

Total vaccine doses administrated over 2000–2020 are aggregated by estimated vaccination state of the target children reached. DRC: Democratic Republic of the Congo. MCV1: the first routine dose of measles-containing vaccine. MCV2: the second routine dose of measles-containing vaccine. SIA: supplementary immunisation activity.

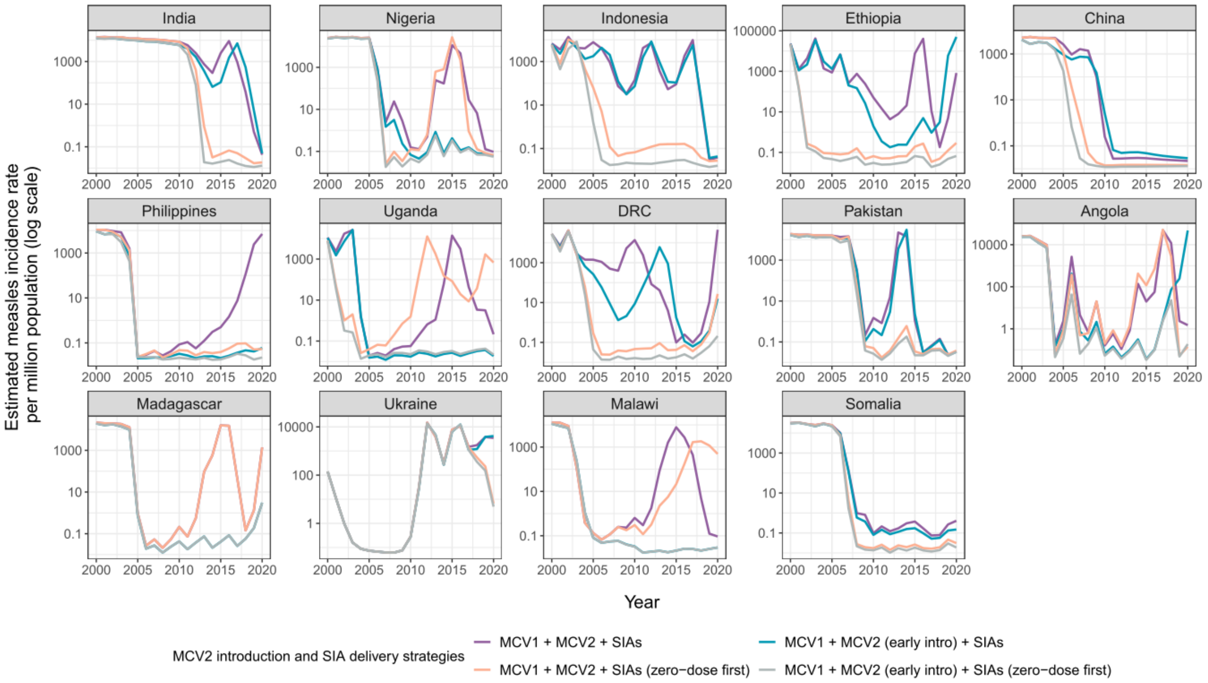

Figure S5. Estimated measles incidence rate (per million) under alternative assumptions of delivering MCV2 and SIAs over 2000–2020.

To estimate the impact of "optimal" vaccination impact (though not changing assumed MCV1 coverage or overall SIA coverage), we combined the alternative assumptions of early MCV2 introduction and SIA dose allocation prioritised for zero-dose populations. Incidence rates for different strategies are in different colours. Note that the y-axis is on the log scale. DRC: Democratic Republic of the Congo. MCV1: the first routine dose of measles-containing vaccine. MCV2: the second routine dose of measles-containing vaccine. SIA: supplementary immunisation activity.

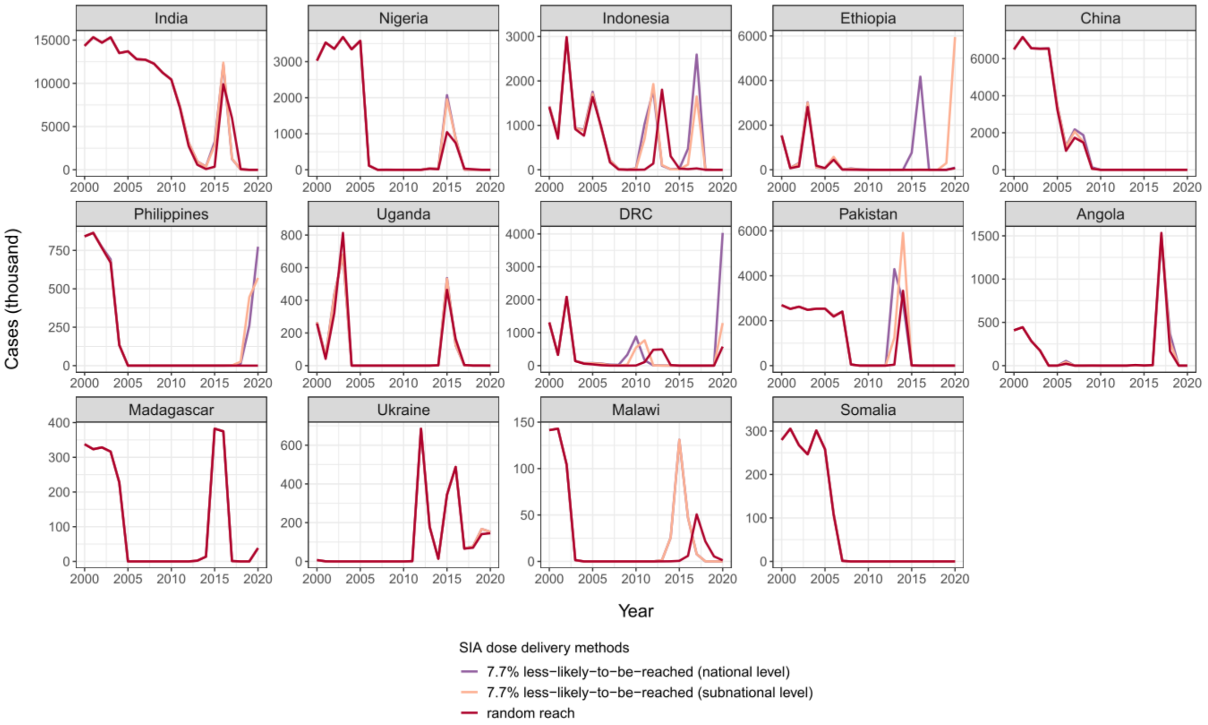

Figure S6. Estimated measles cases (thousands) under different assumptions of SIA dose distribution over 2000–2020.

This figure shows estimated measles cases using three SIA dose delivery methods based on historical coverage of MCV1, MCV2, and SIAs over 2000–2020. In the main analysis, we used the national SIA coverage and assumed SIA doses to be distributed equally to the zero-dose and already-vaccinated populations except for 7.7% of children who are considered less likely to be reached under current immunisation programmes. Alternatively, distributing SIA doses at the subnational level while holding the 7.7%-less-likely-to-be-reach assumption would result in fewer estimated measles cases. Random distribution of SIA doses leads to the lowest burden among the three delivery methods due to better targeting the zero-dose population. DRC: Democratic Republic of the Congo. MCV1: the first routine dose of measles-containing vaccine. MCV2: the second routine dose of measles-containing vaccine. SIA: supplementary immunisation activity.

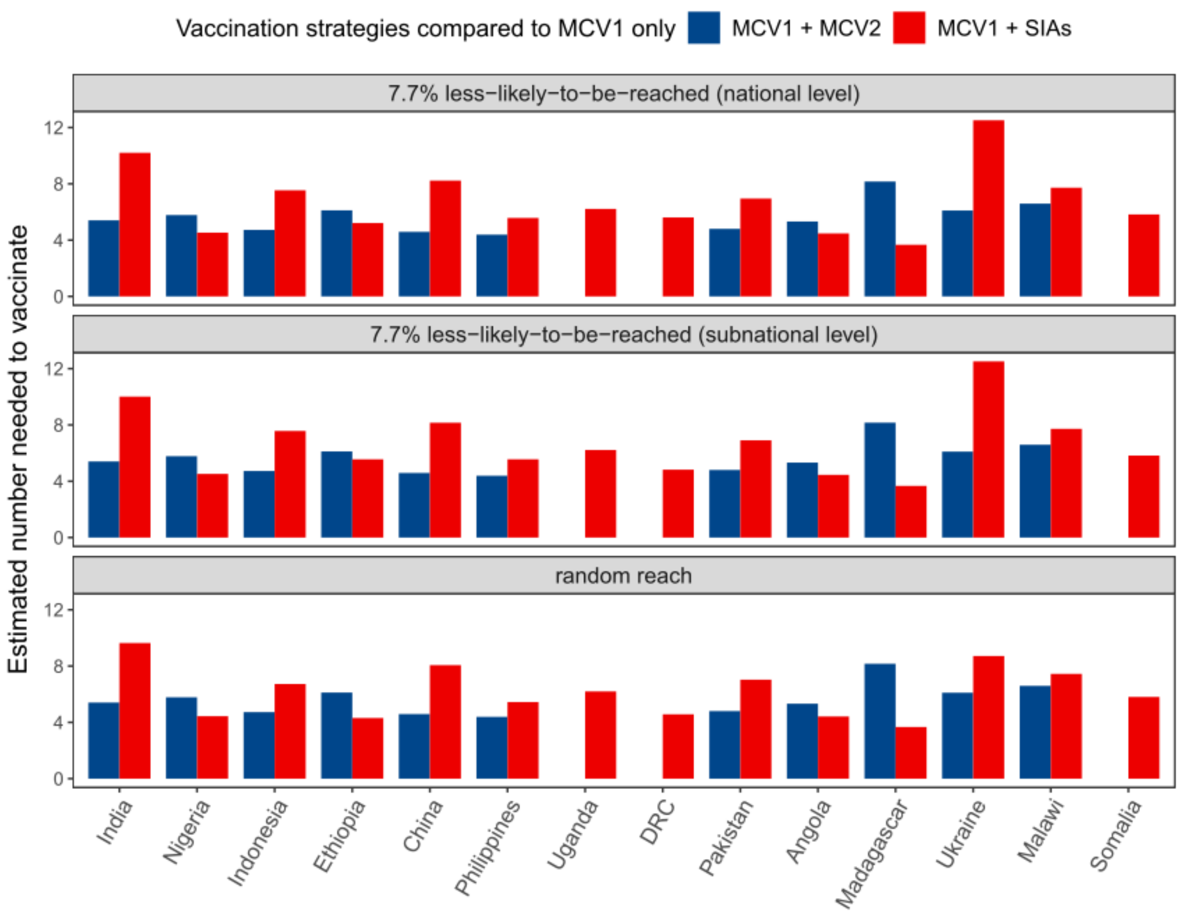

Figure S7. Estimated number needed to vaccinate to avert a measles case under different assumptions of SIA dose distribution over 2000–2020.

The number needed to vaccinate is calculated by dividing the number of additional doses by the number of averted measles cases. The comparator vaccination strategy of MCV1 only was used. DRC: Democratic Republic of the Congo. MCV1: the first routine dose of measles-containing vaccine. MCV2: the second routine dose of measles-containing vaccine. SIA: supplementary immunisation activity.

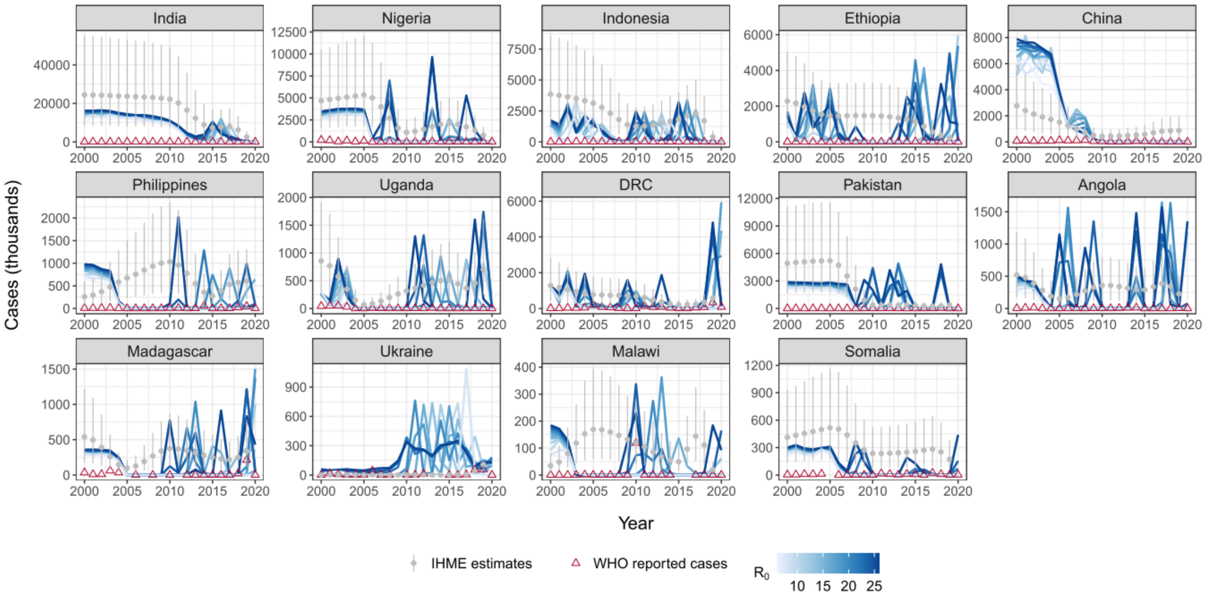

Figure S8. Estimated measles cases and reported cases under different values of R_0 over 2000–2020.

Blue solid lines denote model estimates of measles cases by different values of R_0, based on historical coverage of MCV1, MCV2, and SIAs over 2000–2020. WHO data for reported measles cases and IHME incidence estimates from the Global Burden of Disease 2019 study are included for comparison. The magnitude and trend of our model burden estimates show similarities to the IHME estimates. However, these case estimates are substantially different from the WHO country notifications, which suffer from underreporting and varying capacity for surveillance and diagnosis over time. DRC: Democratic Republic of the Congo. IHME: Institute for Health Metrics and Evaluation. MCV1: the first routine dose of measles-containing vaccine. MCV2: the second routine dose of measles-containing vaccine. SIA: supplementary immunisation activity.
